# Supplementary material for: Overlapping cell population expression profiling and regulatory inference in C. elegans
Source: BMC Genomics. 2016 Feb 29;17:159. doi: 10.1186/s12864-016-2482-z (PMC4772325; doi:10.1186/s12864-016-2482-z)
Supplement: Additional file 13: — Web supplement. (DOC 21 kb) [file 12864_2016_2482_MOESM13_ESM.zip › sortWeb/index.html]

Web supplement to

# Overlapping cell population expression profiling and regulatory inference in *C. elegans*

## Cluster overview

This is an overview of the expression
pattern of each cluster.

## Expression browser

(Note: this requires a relatively recent browser which supports
HTML5.)

The cluster browser provides a way to
find genes with expression similar to a gene of interest. It shows a
gene's expression, and the expression pattern of other genes, in
decreasing order of correlation.

To center the view on a gene's
expression pattern, type its name into the text box, and click
“Submit” (or press “Enter”.) The page will update, so that
the top row is that gene's expression. From here:

- clicking on a gene's name will
  recenter the view on that gene

  - clicking on the description field
    will go to the Wormbase entry for that gene (even if the description
    is blank)

    - clicking on the cluster number
      will go a summary of what was enriched in a particular cluster.

This page has been
tested on Firefox and Chrome (on Windows 7, Mac OS X, and Ubuntu Linux),
Safari version 7 (on Mac OS X), and
Internet Explorer 11 (on Windows 7). Updates to this will be available
from the
Murray lab publications page.
